# Alkaline ceramidase 3 deficiency aggravates colitis and colitis-associated tumorigenesis in mice by hyperactivating the innate immune system

**DOI:** 10.1038/cddis.2016.36

**Published:** 2016-03-03

**Authors:** K Wang, R Xu, A J Snider, J Schrandt, Y Li, A B Bialkowska, M Li, J Zhou, Y A Hannun, L M Obeid, V W Yang, C Mao

**Affiliations:** 1 Department of Medicine, State University of New York at Stony Brook University, Stony Brook, NY, USA; 2 Stony Brook Cancer Center, Stony Brook, NY, USA; 3 Department of Hepatobiliary Surgery, Nanfang Hospital, Southern Medical University, Guangzhou, China; 4 Northport Veterans Affairs Medical Center, Northport, NY, USA; 5 Department of Gastroenterology, Nanfang Hospital, Southern Medical University, Guangzhou, China

## Abstract

Increasing studies suggest that ceramides differing in acyl chain length and/or degree of unsaturation have distinct roles in mediating biological responses. However, still much remains unclear about regulation and role of distinct ceramide species in the immune response. Here, we demonstrate that alkaline ceramidase 3 (Acer3) mediates the immune response by regulating the levels of C_18:1_-ceramide in cells of the innate immune system and that Acer3 deficiency aggravates colitis in a murine model by augmenting the expression of pro-inflammatory cytokines in myeloid and colonic epithelial cells (CECs). According to the NCBI Gene Expression Omnibus (GEO) database, ACER3 is downregulated in immune cells in response to lipopolysaccharides (LPS), a potent inducer of the innate immune response. Consistent with these data, we demonstrated that LPS downregulated both Acer3 mRNA levels and its enzymatic activity while elevating C_18:1_-ceramide, a substrate of Acer3, in murine immune cells or CECs. Knocking out *Acer3* enhanced the elevation of C_18:1_-ceramide and the expression of pro-inflammatory cytokines in immune cells and CECs in response to LPS challenge. Similar to *Acer3* knockout, treatment with C_18:1_-ceramide, but not C_18:0_-ceramide, potentiated LPS-induced expression of pro-inflammatory cytokines in immune cells. In the mouse model of dextran sulfate sodium-induced colitis, Acer3 deficiency augmented colitis-associated elevation of colonic C_18:1_-ceramide and pro-inflammatory cytokines. Acer3 deficiency aggravated diarrhea, rectal bleeding, weight loss and mortality. Pathological analyses revealed that Acer3 deficiency augmented colonic shortening, immune cell infiltration, colonic epithelial damage and systemic inflammation. Acer3 deficiency also aggravated colonic dysplasia in a mouse model of colitis-associated colorectal cancer. Taken together, these results suggest that Acer3 has an important anti-inflammatory role by suppressing cellular or tissue C_18:1_-ceramide, a potent pro-inflammatory bioactive lipid and that dysregulation of ACER3 and C_18:1_-ceramide may contribute to the pathogenesis of inflammatory diseases including cancer.

Ceramides are the central lipid in the metabolic network of sphingolipids, and are generated through the *de novo*, catabolic and salvage pathways.^1^ In the *de novo* pathway, ceramides are synthesized through multiple steps catalyzed sequentially by serine palmitoyltransferase (SPT), keto-dihydrosphingosine reductase, (dihydro)ceramide synthases (CerSs) and dihydroceramide desaturases. In the catabolic pathways, ceramides are derived from the hydrolysis of sphingomyelins by sphingomyelinases (SMases) or the hydrolysis of glycosphingolipids. In the salvage pathway, ceramides are synthesized from sphingosine (SPH) and fatty acyl-CoA by CerSs. As CerSs (CerS1-6) have distinct specificity toward acyl-CoA chain length and degree of unsaturation, ceramides with various acyl-chains are found in mammalian cells. Upon generation, ceramides can be hydrolyzed by five ceramidases encoded by five distinct genes (*ASAH1*, *ASAH2*, *ACER1*, *ACER2* and *ACER3*). These ceramidases vary in pH optimum for catalytic activity, tissue distribution, cellular localization and substrate specificity,^2^ allowing for regulation of specific ceramides in a cell- or tissue-specific manner.

Recent studies have implicated ceramides in regulating the innate immune response. Sakata *et al.*^3^ demonstrated that lipopolysaccharides (LPS), a potent inducer of the innate immune response, increases C_16_-ceramide by activating acid SMase and that inhibition of SMase attenuates LPS-induced production of pro-inflammatory cytokines in THP-1 macrophages. Andreyev *et al.*^4^ found that ceramides are increased by Toll-like receptor 4 (TLR4)-specific LPS in RAW 264.7 macrophages. Schilling *et al.*^5^ revealed that LPS and palmitic acid synergistically increase C_16_-ceramide in primary mouse peritoneal macrophages (PMs) by activating *de novo* biosynthesis of ceramides and that inhibiting the C_16_-ceramide increase attenuates LPS-induced production of TNF-*α* and IL-1*β* in PMs. A recent study found that LPS increases ceramides in Raw 264.7 macrophages through nuclear factor kappa B (NF-*κ*B)-dependent upregulation of SPT long chain base subunit 2 Sptlc2, a regulator of SPT.^6^ These results suggest that ceramides mediate the immune response in part by enhancing the production of pro-inflammatory cytokines in innate immune cells.

Emerging evidence suggests that dysregulation in the innate immune response in inflammatory bowel disease (IBD) contributes to the pathogenesis of the disease.^7^ Consistent with the role of ceramides in potentiating the innate immune response, several studies found that ceramides may have a role in the pathogenesis of IBD. Sakata *et al.*^3^ demonstrated that blocking the generation of ceramides with the SMase inhibitor hinders mouse colitis. Fischbeck *et al.*^8^ showed that increasing ceramides in the gut by supplying mice with dietary sphingomyelins, a precursor of ceramides, aggravates mouse colitis. These results suggest that increased levels of ceramides may contribute to the pathogenesis of IBD.

Although the role of ceramides and their generating enzymes in the innate immune response have been well studied, much remains unclear about the role of ceramidases involved in the catabolism of ceramides in this biological response. In this study, we investigated the role of alkaline ceramidase 3 (ACER3)/Acer3 and its substrates in immune response. We demonstrated that Acer3 is downregulated, whereas its substrate, C_18:1_-ceramide, is upregulated in murine immune cells and colonic epithelial cells (CECs) during the innate immune response to LPS. Using *Acer3* null mice (Acer3^−/−^) and their wild-type (Acer3^+/+^) littermates, we further discovered that the inverse regulation of Acer3 and C_18:1_-ceramide potentiates LPS-induced production of pro-inflammatory cytokines in innate immune cells. More importantly, we found that Acer3 deficiency aggravates dextran sulfate sodium (DSS)-induced colitis and colitis-associated colorectal cancer (CAC) in a murine model. These findings indicate that Acer3/ACER3 and C_18:1_-ceramide are novel modulators in the innate immune response and that their dysregulation may contribute to the pathogenesis of inflammatory diseases.

## Results

### Acer3 downregulation mediates LPS-induced upregulation of C_18:1_-ceramide in immune cells and CECs

The mechanism by which LPS regulates ceramide metabolism is still unclear. Our previous studies have demonstrated that the human ACER3 (ref. 9) and its mouse counterpart Acer3 (ref. 10) regulate unsaturated-long-chain ceramides (ULCCs), including C_18:1_-ceramide and C_20:1_-ceramide. To investigate if ACER3 mediates the innate immune response, we first determined if ACER3 is regulated by LPS in immune cells by analyzing the NCBI Gene Expression Omnibus (GEO) database. *In silico* analyses revealed that *ACER3* mRNA levels are downregulated in human macrophages,^11, 12^ monocytes^13^ and dendritic cells^14^ upon LPS stimulation (Supplementary Figure S1A).

**Figure 1:**
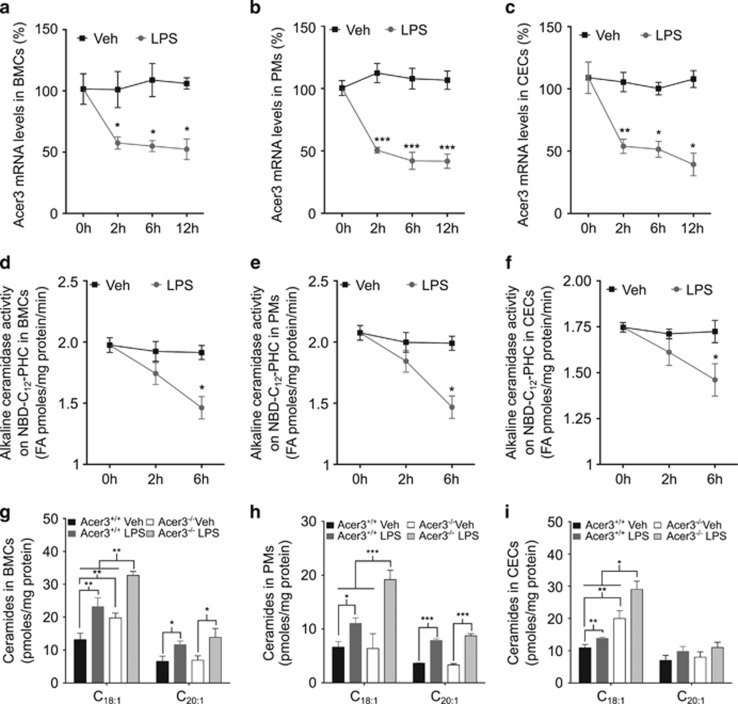
Acer3 downregulation enhances LPS-induced upregulation of C_18:1_-ceramide in immune cells and CECs. (**a–c**), BMCs (**a**), PMs (**b**) and CECs (**c**) were isolated from C57BL/6 mice as described in Materials and methods section. BMCs were plated at a density of 10^6^ cells per well in six-well culture plates and immediately treated with 50 ng/ml LPS or Veh (PBS) for the indicated times. PMs and CECs were plated at the same cell density in regular and Matrigel-coated six-well plates, respectively, for 60 min before being treated with LPS or PBS. Total RNAs were isolated from the above treated cells and *Acer3* mRNA levels were measured by qPCR analyses. Data are expressed as percent changes of *Acer3* mRNA levels in LPS-treated cells over those in Veh-treated cells. (**d**–**f**), BMCs (**d**), PMs (**e**) and CECs (**f**) were treated with LPS or PBS as above. Total membranes were isolated from cells and were assayed for alkaline ceramidase. (**g–i**) BMCs (**g)**, PMs (**h**) and CECs (**i**) from Acer3^+/+^ and Acer3^−/−^ mice were plated at 10^8^ cells per well as above and treated with 50 ng LPS or PBS for 6 h before they were subjected to LC-MS/MS for ceramide levels. Data represent mean±S.D. from three independent experiments. *P<0.05, **P<0.01, ***P<0.001

To facilitate our studies on the role of ACER3 in immune response using mouse models, we examined Acer3 expression in response to LPS in murine immune cells. We found that LPS downregulated *Acer3* mRNA levels and enzymatic activity in blood mononuclear cells (BMCs) (Figures 1a and d) and PMs (Figures 1b and e) isolated from WT C57BL6/J mice. Certain epithelial cells were shown to have an important role in the innate immune response,^15^ including CECs.^7^ We found that LPS also downregulated *Acer3* mRNA levels (Figure 1c) and its enzymatic activity (Figure 1f) in CECs. Taken together, these results suggest that LPS suppresses ACER3/Acer3 expression in immune cells and CECs.

To determine if Acer3 downregulation had a role in elevating ceramides in response to LPS stimulation, we determined the levels of ceramides in LPS-stimulated BMCs, PMs or CEC isolated from Acer3^−/−^ mice and their WT littermates (Acer3^+/+^). Indeed, liquid chromatography tandem mass spectrometry (LC-MS/MS) analyses found that LPS increased various ceramides in BMCs (Figure 1g and Supplementary Figure S2A), PMs (Figure 1h and Supplementary Figure S2B) or CECs (Figure 1i and Supplementary Figure S2C) and that *Acer3* knockout only enhanced the LPS-induced increase of C_18:1_-ceramide (Figures 1g–i), but not other ceramide species, SPH or sphingosine-1-phosphate (S1P) in these cells (Supplementary Figures S2A and D). These results suggest that Acer3 downregulation is important for LPS-induced increase of C_18:1_-ceramide in immune cells and CECs.

### Loss of Acer3 or treatment of C_18:1_-ceramide promotes pro-inflammatory cytokine expression in immune cells and CECs upon LPS stimulation

**Figure 2:**
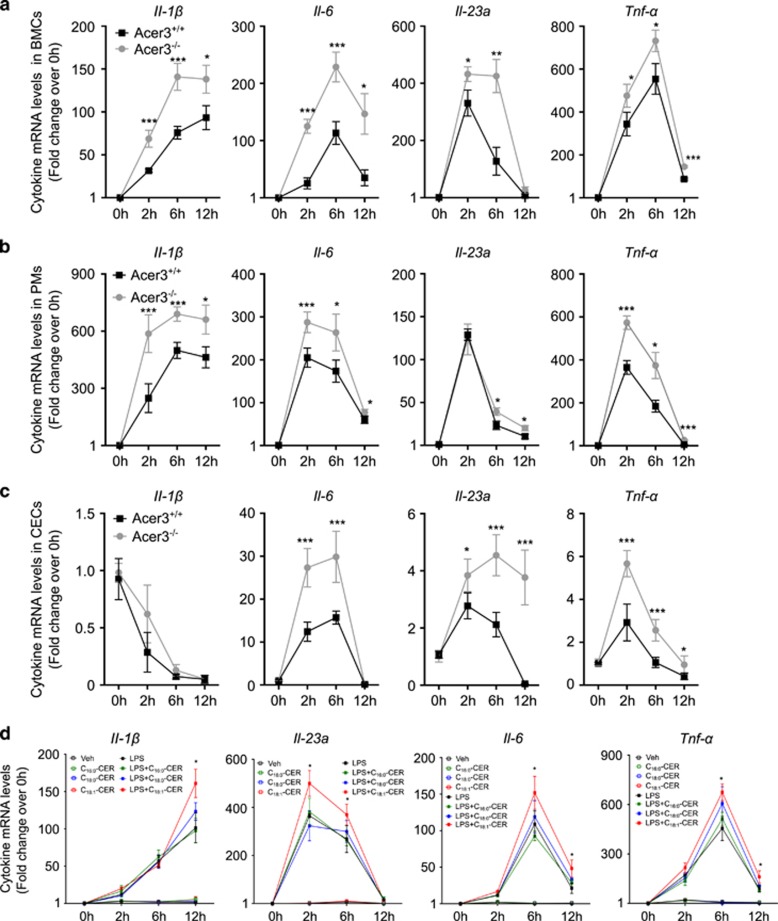
Acer3 deficiency promotes LPS-induced expression of inflammatory cytokines in mouse immune cells and CECs. (**a–c**) BMCs (**a**), PMs (**b**) and CECs (**c**) were isolated from Acer3^+/+^ or Acer3^−/−^ mice and treated with LPS or PBS for indicated times before the mRNA levels of *Il-1β, Il-6, Il-23, Tnf-α* were determined by qPCR with *β*-actin as a reference gene. The mRNA levels are expressed as fold changes induced by LPS treatment over Veh treatment. (**d**) Mouse BMCs were treated with LPS (50 ng/ml), C_16_-ceramide (0.5 *μ*M) alone or plus LPS, C_18_-ceramide (0.5 *μ*M) alone or plus LPS, C_18:1_-ceramide (0.5 μM) alone or plus LPS, or Veh (PBS or ethanol) for 2, 6 and 12 h before the mRNA levels of *Il-1β, Il-23, Il-6* or *Tnf*-*α* were determined by qPCR analyses with *β-*actin as a reference gene. Data are expressed as fold change relative to the basal mRNA levels at time 0 h. Data represent mean±S.D. from three independent experiments. *P<0.05, **P<0.01, ***P<0.001

Ceramides have been shown to regulate pro-inflammatory cytokines in response to LPS.^3, 16^ Having demonstrated that Acer3 downregulation mediates LPS-induced elevation of C_18:1_-ceramide, we investigated if loss of Acer3 affected the expression of pro-inflammatory cytokines in BMCs, PMs and CECs stimulated by LPS. In BMCs, Acer3 deficiency enhanced and prolonged LPS-induced increases in the mRNA levels of *Il-1β*, *Il-6*, *Il-23a* and *Tnf-α* (Figure 2a). In PMs, Acer3 deficiency enhanced LPS-induced increases in the mRNA levels of *Il-1β*, *Il-6* and *Tnf-α*, and to a lesser extent, *Il-23a* (Figure 2b). In CECs, Acer3 deficiency augmented LPS-induced increases in the mRNA levels of *Il-6*, *Il-23a* and *Tnf-α* but not *Il-1β* (Figure 2c). Acer3 deficiency did not affect the basal mRNA levels of these pro-inflammatory cytokines (Supplementary Figures S3A, S3B, and S3C). These results suggest that Acer3 deficiency potentiates LPS-induced upregulation of pro-inflammatory cytokines in immune cells and CECs.

As Acer3 deficiency potentiated LPS-induced increases of C_18:1_-ceramide and pro-inflammatory cytokines in cells, we investigated if treatment with C_18:1_-ceramide mimicked Acer3 deficiency and potentiated LPS-induced pro-inflammatory cytokines in BMCs. Indeed, we found that exogenous C_18:1_-ceramide but not its saturated analog C_18:0_-ceramide or C_16:0_-ceramide significantly enhanced the LPS-induced upregulation of pro-inflammatory cytokines, including *Il-1β*, *Il-23a*, *Il-6* and *Tnf-α* (Figure 2d) in BMCs. These results suggest that Acer3 deficiency potentiates LPS-induced expression of pro-inflammatory cytokines in BMCs likely by upregulating C_18:1_-ceramide.

### Acer3 is downregulated, whereas C_18:1_-ceramide is upregulated in the murine colon tissues during colitis

**Figure 3:**
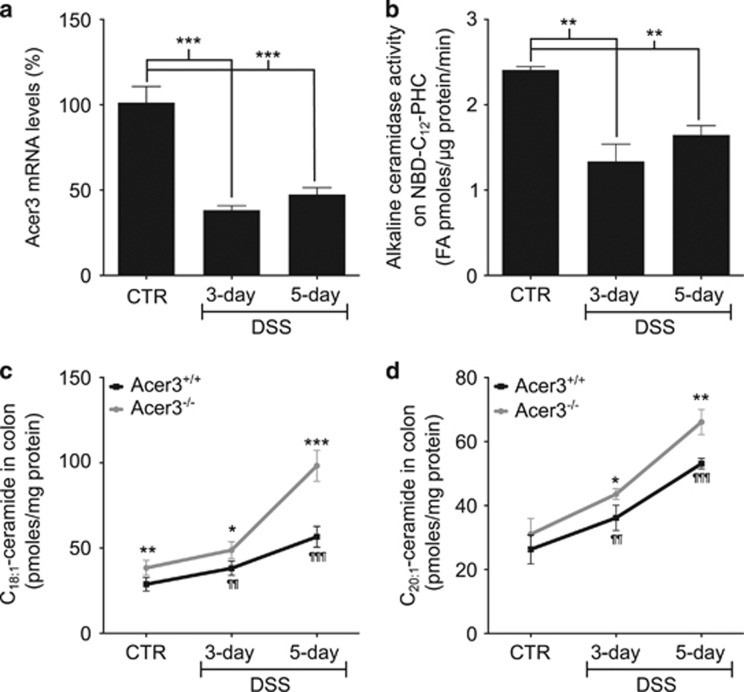
Acer3 deficiency enhances the elevation of C_18:1_-ceramide in mouse colon upon colitis induction. (**a** and **b**) RNA and total membranes were prepared from colons that were dissected from WT mice treated with DSS for 3 or 5 days. The total RNA and membranes were subjected to qPCR analyses for *Acer3* mRNA levels (**a**) and Acer3 activity assays (**b**), respectively. For qPCR, *β-*actin gene was used as a reference gene. Data are expressed as percent changes in DSS-treated mice *versus* CTR mice. (**c** and **d**) Colon tissues were collected from Acer3^+/+^ and Acer3^−/−^ mice on DSS or regular water for 5 days and the levels of C_18:1_-ceramide (**c**) and C_20:1_-ceramide (**d**) were determined by LC-MS/MS. Data represent mean±S.D., *n*=4–6. *P<0.05, **P<0.01, ***P<0.001, Acer3^+/+^ mice *versus* Acer3^−/−^ mice. *P*<0.01, *P*<0.001, Acer3^+/+^ CTR mice *versus* Acer3^+/+^ DSS-treated mice

IBD is manifested by hyperactive immune response in colon.^7^ It was shown that this hyperactive immune response is partially attributed to the translocation of microbiomes and/or their products, including LPS, from the colon lumen to mucosa and submucosal layers.^17, 18, 19, 20^ As Acer3 and C_18:1_-ceramide regulated LPS-induced expression of pro-inflammatory cytokines, we investigated whether ACER3/Acer3 and C_18:1_-ceramide had a role in the pathogenesis of IBD. To this end, we used a murine model of colitis induced by DSS. First, we determined if Acer3 and its regulated C_18:1_-ceramide were altered in mouse colons in response to DSS-induced colitis. We showed that DSS-induced pathologic manifestations of colitis (Supplementary Figure S4). Upon treatment with DSS, both *Acer3* mRNA levels (Figure 3a) and its enzymatic activity (Figure 3b) were significantly decreased in the colon. LC-MS/MS showed that DSS treatment increased various ceramides including C_18:1_ and C_20:1_-ceramides in the colon and that *Acer3* deficiency enhanced the increase of C_18:1_-ceramide (Figure 3c), and to a lesser extent, C_20:1_-ceramide (Figure 3d), but not other ceramide species, SPH or S1P (Supplementary Figures S2E and 2F). These data suggest that Acer3 deficiency mainly enhances the colitis-associated elevation of colonic C_18:1_-ceramide.

### Acer3 deficiency enhances upregulation of pro-inflammatory cytokines in colon upon colitis induction

**Figure 4:**
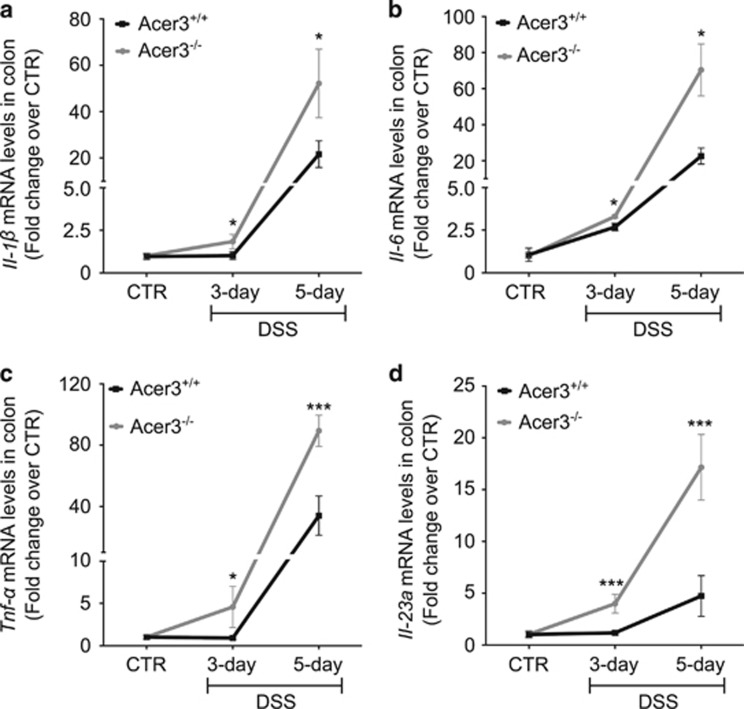
Acer3 deficiency enhances the upregulation of inflammatory cytokines in colon upon colitis induction. (**a–d**) Total RNA was extracted from colon tissues of mice treated with DSS for indicated times, and the mRNA levels of *Il-1β* (**a**), *Il-6* (**b**)*, Tnf-α* (**c**) or *Il-23a* (**d**) were determined by qPCR with *β*-actin as a reference gene. Fold change of mRNA levels in DSS-treated mice *versus* the CTR mice were determined by ΔΔCT method. Data represent mean±S.D., *n*=5. *P<0.05, ***P<0.001

As Acer3 deficiency promoted LPS-induced cytokine upregulation in cells, we determined if Acer3 deficiency also affected inflammatory cytokines in the colon tissues of mice with colitis. The results revealed that *Acer3* knockout significantly augmented the colitis-induced increases in the mRNA levels of *Il-1β*, *Il-6*, *Tnf-α* and *Il-23a* (Figures 4a–d) in colon, although Acer3 deficiency did not affect the basal mRNA levels of these inflammatory cytokines (Supplementary Figure S3D). These results suggest that *Acer3* knockout enhances the expression of pro-inflammatory cytokines in colon upon induction of colitis.

### Acer3 deficiency exaggerates local inflammatory manifestations during acute colitis

**Figure 5:**
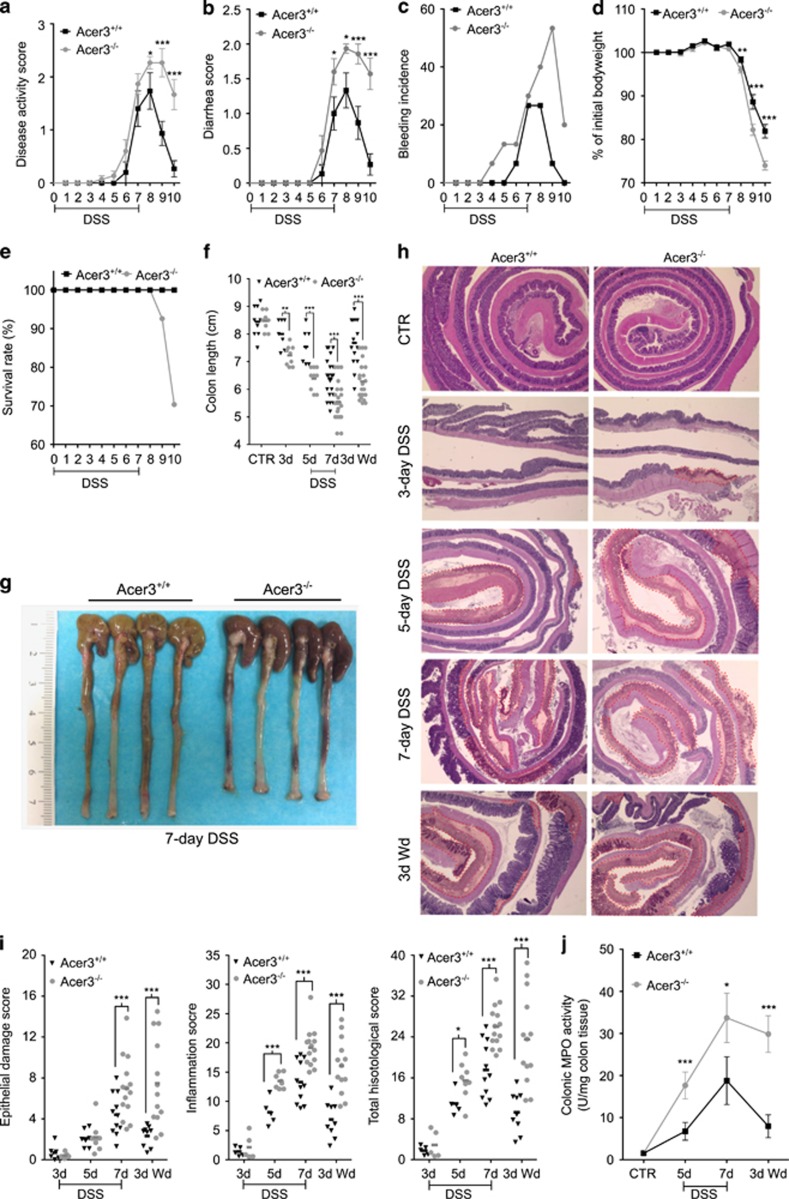
Acer3 deficiency exaggerates local and systemic inflammatory manifestations during acute colitis. (**a–e**) Gender-matched Acer3^+/+^ and Acer3^−/−^ mice at 6 weeks of age were on drinking water containing 2.5% w/v DSS for 7 days. Disease activity (**a**), diarrhea score (**b**), rectal bleeding incidence (**c**), bodyweight loss (**d**) or survival rate (**e**) were monitored daily until 3 days after DSS withdrawal (3d Wd), *n*=40. (**f–j**) Mice were killed after 3, 5 or 7-day DSS treatment or 3 days post 7-day DSS treatment (3d Wd), and the colons were removed from the animals. The lengths of the colons were measured (**f** and **g**). The colons were rolled, sectioned and stained with H&E (**h**), and damaged areas were highlighted with red dotted lines. For mice treated with DSS for 3 days, images were captured from the distal colon only. The scores of inflammation (**i**) and epithelial damage (**i**) were separately obtained as described in Materials and methods section, and were summed up into total histologic scores (**i**). Freshly dissected colon tissues were measured for MPO activity (**j**). Data in (**a**), (**b**) and (**d**) represent mean±S.E.M., *n*=40; data in (**j**) represent mean±S.D., *n*=5. *P<0.05, **P<0.01, ***P<0.001

**Figure 6:**
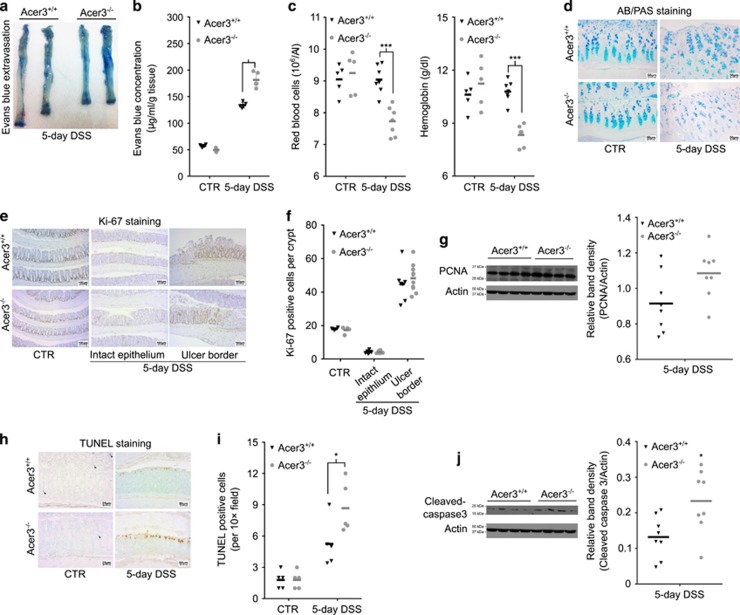
Acer3 deficiency exaggerates vascular leakage, mucous depletion and apoptosis in the mouse model of colitis. (**a–c**) Acer3^+/+^ and Acer3^−/−^ mice were on DSS-containing or regular water for 5 days, and were subjected to vascular permeability assays (**a** and **b**) RBC counts and hemoglobin measurements (**c**). (**d**) Colonic sections from Acer3^+/+^ and Acer3^−/−^ mice on DSS-containing or regular water for 5 days were co-stained with AB and APS as described under Materials and methods section. (**e** and **f**) Colonic sections from Acer3^+/+^ and Acer3^−/−^ mice on DSS-containing or regular water were stained with anti-Ki-67 antibody (**e**) and Ki-67-positive cells were counted (**f**) to evaluate proliferation. (**g**) Colons from Acer3^+/+^ and Acer3^−/−^ mice on DSS-containing or regular water were subjected to western blot analyses with antibody against PCNA or *β*-actin (a protein loading control), and PCNA level was quantified by densitometry. (**h** and **i**) Colonic sections were subjected to TUNEL staining (**h**) and TUNEL-positive cells were numerated (**i**). (**j**) Tissue homogenates prepared as in (**g**) were subjected to western blot analyses with anti-caspase-3 antibody, and the cleaved capsase-3 was quantified by densitometry. Images in (**a**), (**d**), (**e**) and (**h**) represent results from one of five pairs of mice. *P<0.05, ***P<0.001

The above results prompted us to investigate whether Acer3 downregulation had a role in pathogenesis of colitis. Colitis characteristics, including bleeding and diarrhea, were monitored in Acer3^−/−^ and Acer3^+/+^ mice during and after colitis induction. We found that compared with Acer3^+/+^ mice, Acer3^−/−^ mice had higher colitis activity scores (Figure 5a) with severe diarrhea (Figure 5b) and bleeding (Figure 5c). After DSS withdrawal, Acer3^−/−^ mice recovered from colitis more slowly than Acer3^+/+^ mice (Figures 5a–c). Correspondingly, Acer3^−/−^ mice also exhibited a greater bodyweight loss (Figure 5d) and higher mortality rates (Figure 5e), although an equal DSS intake was observed between Acer3^−/−^ and Acer3^+/+^ mice (Supplementary Figure S5). After necropsy, examination of the colon found that during the acute colitis Acer3^−/−^ mice had earlier and greater colonic shortening, compared with Acer3^+/+^ mice (Figures 5f and g), higher scores of colonic epithelial damage and inflammatory infiltration (Figures 5h and i), and a greater colonic myeloperoxidase (MPO) activity (Figure 5j). Vascular leakage examination by extravasation of Evan's blue dye (EBD)^21, 22^ revealed a significantly greater extravasation of EBD in Acer3^−/−^ mice compared with Acer3^+/+^ mice (Figures 6a and b). Complete blood cell count (CBC) revealed a greater reduction in red blood cells (RBCs) and hemoglobin in Acer3^−/−^ mice than in Acer3^+/+^ mice (Figure 6c), suggesting that Acer3 deficiency worsens colonic bleeding during acute colitis. PAS/AB staining showed that upon colitis induction, Acer3^−/−^ mice had a higher loss of mucous-producing epithelial cells, a characteristic lesion of DSS-induced colitis, than Acer3^+/+^ mice (Figure 6d). Acer3 deficiency did not affect epithelial cell proliferation (Figures 6e–g) but worsened epithelial apoptosis during acute colitis (Figures 6h–j). These results collectively suggest that Acer3 deficiency exaggerates colitis in mice.

### Acer3 deficiency exaggerates systemic inflammatory manifestations during acute colitis

**Figure 7:**
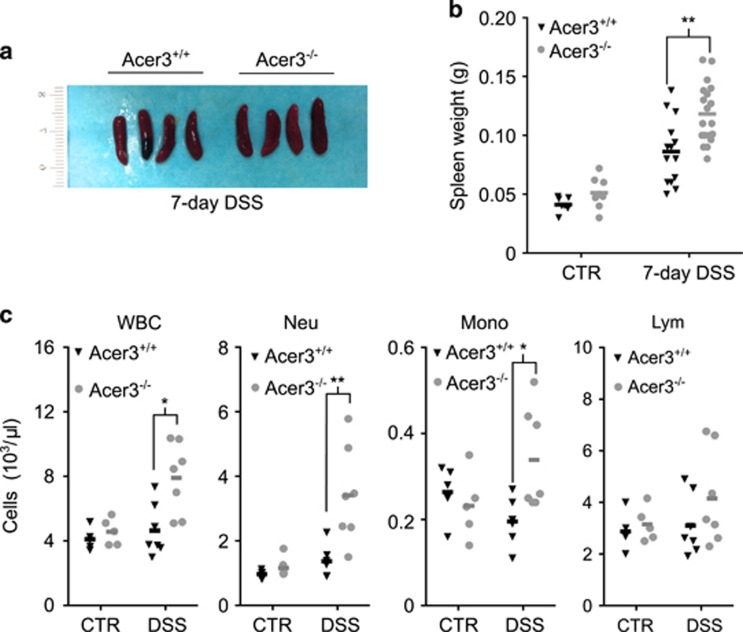
Acer3 deficiency exaggerates systemic inflammatory manifestations during acute colitis. (**a–c**) Acer3^+/+^ or Acer3^−/−^ mice on DSS or regular water for 7 days before spleens were dissected, imaged (**a**) and weighted (**b**). From these mice, blood was collected and subjected to complete blood counts for neutrophil (Neu), monocyte (Mono), lymphocyte (Lym) and WBC counts (**c**). *P<0.05, **P<0.01

As systemic inflammatory manifestations, including white blood cell elevation and spleen enlargement, have been observed in acute DSS-induced colitis,^23, 24, 25^ we examined Acer3 deficiency in the systemic inflammatory response during colitis. At necropsy, Acer3^−/−^ mice had a greater spleen enlargement (Figures 7a and b) and higher white blood cell counts than Acer3^+/+^ mice after 7-day DSS treatment (Figure 7c). Acer3 deficiency did not affect circulating immune cell counts at baseline (Figure 7c). These results show that Acer3 deficiency sensitizes mice to colitis-induced systemic inflammation.

### Loss of Acer3 promotes colitis-associated dysplasia progression

**Figure 8:**
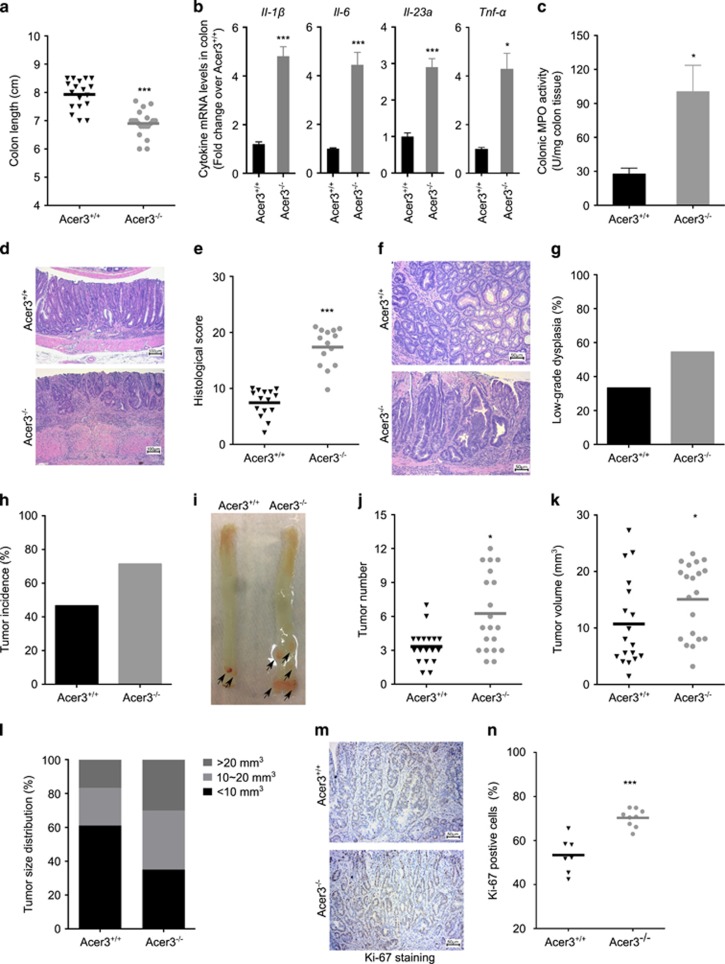
Acer3 deficiency promotes colitis-associated dysplasia progression. (**a**–**e**) Acer3^+/+^ and Acer3^−/−^ mice were subjected to CAC, and pathological parameters of colitis were examined, including colon lengths (**a**), mRNA levels of colonic cytokines (*Il-1β, Il-6, Il-23a* and *Tnf-α*) (**b**), MPO activity (**c**), inflammatory infiltration and epithelial loss (**d**) and histological scores (**e**). (**f**–**l**) Colons were dissected from Acer3^+/+^ and Acer3^−/−^ mice subjected a mode of CAC. The colons were opened along the mesenteric side and analyzed for colonic dysplasia (**f**), incidence of colonic dysplasia (**g**), tumor incidence in colon (**h** and **i**), average tumor number (**j**), average tumor size (**k**) and tumor size distribution (**l**), *n*=19-20. (**m** and **n**) Colon sections were immunostained with anti-Ki67 antibody and imaged (**m**) and Ki-67-positive cells were enumerated. Data in (**b**) and (**c**) represent mean±S.D., *n*=5. *P<0.05, ***P<0.001

The causal link between colitis and colorectal cancer has been well established.^26^ Based on the finding that Acer3 deficiency results in increased colitis in a murine model, we utilized a murine model of colorectal carcinogenesis^27^ to examine the role of Acer3 in CAC. Acer3^−/−^ mice were vulnerable to DSS-induced colitis; therefore, we subjected the mice to a single injection of azoxymethane (AOM) followed by a single 7-day course of DSS.^27^ In this model, Acer3^−/−^ mice consistently exhibited a higher mortality rate than Acer3^+/+^ mice (Supplementary Figure S6A). Acer3^−/−^ mice that survived DSS treatment showed more severe colitis, as evidenced by a greater colon shortening (Figure 8a), higher levels of inflammatory cytokines (*Il-1β, Il-6, Il-23a* and *Tnf-α*) (Figure 8b), higher MPO activity (Figure 8c) and more severe pathological manifestations (Figures 8d and e). We also found that Acer3^−/−^ mice had a greater propensity to develop low-grade dysplasia in the inflamed epithelium (Figures 8f and g) and exhibited a higher tumor incidence (Figure 8h) and greater tumor multiplicity (Figures 8i and j) compared with Acer3^+/+^ mice. Although Acer3 deficiency did not affect average tumor size (Figure 8k), they harbored large tumors more frequently than Acer3^+/+^ mice (Figure 8l). Ki-67 staining revealed a higher degree of cell proliferation in the tumors in Acer3^−/−^ mice than in Acer3^+/+^ mice (Figures 8m and n). Acer3^−/−^ mice also had higher WBC counts and greater splenomegaly than Acer3^+/+^ mice (Supplementary Figure S6B), suggesting that Acer3 deficiency prolongs systemic inflammatory response in mice during carcinogenesis. Taken together, these data highlight an important role for Acer3 in inhibiting persistent colonic and systemic inflammation and inflammation-associated dysplasia.

## Discussion

In this study, we demonstrate that Acer3 is an important inflammatory modulator that keeps the innate immune response in check by maintaining the pro-inflammatory bioactive C_18:1_-ceramide at low levels in immune cells and tissues. LPS has been shown to upregulate ceramide-generating enzymes,^28^ including acid SMase,^3^ neutral SMase^29^ and SPT^6^ in macrophages. However, whether LPS also regulates ceramidases responsible for the catabolism of ceramides in the immune system remains unclear. *In silico* analyses showed that ACER3 mRNA levels are markedly downregulated in immune cells in response to LPS^11, 12, 13, 14^ (Supplementary Figure S1A). Consistent with these data, we found that LPS also markedly downregulated Acer3 in mouse PMs, BMCs and CECs (Figures 1a–f). More importantly, we revealed that Acer3 was downregulated in colon during acute colitis (Figures 3a and b). During colitis, both immune cells and CECs could be exposed to LPS released from gut microbes;^17, 18, 20^ therefore, colitis-associated downregulation of Acer3 in the colon may be triggered by LPS. Our *in vitro* findings demonstrated that LPS suppressed Acer3 in both immune cells and CECs, so we postulated that the downregulation of Acer3 in colon may also occur in these cell types during colitis. The mechanism by which LPS downregulates ACER3 or Acer3 is under investigation.

LPS has been shown to increase ceramides by activating either *de novo* synthesis^30, 31, 32, 33^ or sphingomyelin degradation.^3, 34, 35, 36, 37, 38, 39^ However, the role of ceramidases in mediating LPS-induced increase of ceramides in the immune system remains unclear. In this study, we found that LPS elevated various ceramides, including C_18:1_-ceramide, in mouse immune cells and CECs *in vitro* with a concomitant downregulation of Acer3, and the increase in C_18:1_-ceramide was enhanced by *Acer3* knockout (Figures 1g–i,Supplementary Figures S2A and S2C). We also showed that C_18:1_-ceramide was also elevated in colon with a concomitant downregulation of Acer3 during colitis induction in WT mice, and the elevation of C_18:1_-ceramide was augmented by Acer3 deficiency (Figures 3c and d). These findings suggest that LPS increases C_18:1_-ceramide by activating its generation while attenuating its degradation.

We recently demonstrated that an accumulation of C_18:1_-ceramide in the central nervous system (CNS) because of Acer3 deficiency led to loss of Purkinje cells, suggesting that C_18:1_-ceramide may be a pro-death bioactive lipid to certain neurons.^10^ However, the role of C_18:1_-ceramide in non-CNS tissues remains largely unclear. Interestingly, C_18:1_-ceramide was recently found to be associated with liver fibrosis progression and poor treatment outcome in patients with hepatitis C virus infection,^40^ suggesting that C_18:1_-ceramide may be involved in immune response in humans. This notion is in line with our findings that upregulation of C_18:1_-ceramide because of Acer3 downregulation potentiates the production of pro-inflammatory cytokines in immune cells and/or tissues (Figures 2 and 4). In both *in vitro* and *in vivo* systems, we observed that the elevation of C_18:1_-ceramide was accompanied with the upregulation of cytokines and that treatment with C_18:1_-ceramide significantly augmented LPS-induced upregulation of inflammatory cytokines (Figure 2d), suggesting a role for C_18:1_-ceramide in mediating the production of cytokines. Interestingly, we found that C_18:1_-ceramide did so more potently than its saturated analog, C_18:0_-ceramide (Figure 2d). Therefore, our studies for the first time demonstrate that C_18:1_-ceramide is a potent pro-inflammatory bioactive lipid that modulates the innate immune response by regulating inflammatory cytokines in innate immune cells. In contrast to C_18:1_-ceramide, several studies demonstrated that a non-endogenous, short-chain ceramide, C_8_-ceramide, inhibits LPS-induced production of cytokines in immune cells.^41, 42^ These results suggest that ceramides with different acyl-chains may have distinct roles in regulating immunity. Indeed, increasing studies suggest that ceramides with different acyl-chains, which were once thought to have the same signaling function, have distinct roles in regulating biological responses, including cell proliferation and survival.^43^

How C_18:1_-ceramide potentiates cytokine expression during innate immune response remains unclear. LPS was shown to induce the translocation of TLR4 and its binding partners to lipid rafts on the plasma membrane and this translocation is thought to be important in activating downstream signaling pathways that mediate cytokine expression.^44, 45^ Ceramides with different acyl-chains were shown to affect the properties of lipid rafts distinctly,^46, 47^ so they may differentially regulate the translocation of TLR4 to lipid rafts and alter downstream signaling. This may explain why C_18:1_-ceramide is distinct in potentiating LPS-induced immune response from other tested ceramide species. This possibility is currently under investigations.

Apart from ceramides, SM derived from ceramides are also implicated in LPS-induced inflammation.^48, 49^ Our previous study demonstrated that *Acer3* knockout caused a slight increase in C_18:1_-SM levels in mouse tissues,^10^ suggesting that part of accumulated C_18:1_-ceramide is converted to C_18:1_-SM. In this study, we found that LPS treatment increased C_18:1_-SM in BMCs and PMs but not CECs and that Acer3 deficiency enhanced the LPS-induced increase of C_18:1_-SM in PMs and CECs (Supplementary Figures S7A and S7B). Interestingly, Acer3 deficiency did not affect colitis-associated increases in this SM species in the colon (Supplementary Figure S7C). These results suggest that Acer3 deficiency worsens colitis and CAC probably not through SMs although Acer3 may regulate the innate immune response in part by controlling C_18:1_-SM in PMs and CECs.

Although cytokines appear to have a major role in driving inflammation in IBD,^7^ the epithelial barrier has also been proven to have a critical role in suppressing intestinal inflammation.^50^ Under basal conditions, Acer3^−/−^ mice did not exhibit decreased intestinal epithelial barrier integrity (Supplementary Figure S3E), and these mice did not develop spontaneous colitis up to 8 months of age (Supplementary Figure S3F). This excludes the possibility that Acer3 deficiency exacerbates colitis by disrupting the epithelial barrier. During acute colitis, we found that Acer3 deficiency worsened epithelial damage (Figures 5h and i) and epithelial apoptosis (Figures 6h–j). The increased apoptosis could be due to the increased TNF-*α*, which is known to induce apoptosis.^51^ Ceramides have been shown to exert an apoptotic effect on CECs during colitis,^8^ so C_18:1_-ceramide accumulated in CECs may also be responsible for increased apoptosis. Based on these observations, we postulated that Acer3 deficiency aggravates colitis by upregulating pro-inflammatory cytokines in myeloid cells and CECs as well as apoptosis of CECs in a C_18:1_-ceramide-dependent manner.

In addition to Acer3/ACER3, two other members, Acer1/ACER1 and Acer2/ACER2, in the alkaline ceramidase family have been found in mice and humans.^52, 53^ ACER1 is unlikely to be involved in the innate immune response as it is not expressed in immune tissues.^53^ Our previous study showed that ACER2 is expressed in various tissues.^52^ Although existing microarray data^11, 12, 13, 14^ did not reveal whether LPS regulates ACER2 in immune cells, we found that Acer2 mRNA levels were upregulated in mouse BMCs and PMs by LPS (Supplementary Figure S1B). We are currently investigating the role of Acer2 in mediating innate immune responses. In addition to the alkaline ceramidases, loss of Asah2, another ceramidase, has been shown to augment the elevation of many ceramide species and aggravate mouse colitis by upregulating Tnf-α and cyclooxygenase-2 expression in colon.^25^ These results together suggest that ceramidases have a key role in preventing hyper-activation of the immune response to the gut microbiota by maintaining the homeostasis of pro-inflammatory ceramides.

Persistent intestinal inflammation has been proven to facilitate colorectal carcinogenesis.^26^ In line with this view, we found that Acer3 deficiency promoted tumorigenesis in murine CAC (Figure 8), suggesting that Acer3 deficiency or an aberrant accumulation of C_18:1_-ceramide promotes colitis-associated dysplasia in colon likely by sustaining colonic inflammation.

In conclusion, for the first time we demonstrate that Acer3 expression has an important role in the innate immune response by regulating the expression of pro-inflammatory cytokines in cells of the innate immune system through C_18:1_-ceramide and that an aberrant accumulation of C_18:1_-ceramide because of Acer3/ACER3 inhibition may contribute the pathogenesis of the inflammatory diseases.

## Materials and Methods

### Mice

Mice were housed under conventional laboratory conditions with constant room temperature (22 °C), humidity level (55%), a 12-h light:12-h dark cycle and food (WF Fisher & Son, Somerville, NJ, USA) and water available *ad libitum*. The Acer3 knockout mouse line was generated as described in our previous studies.^10^ Briefly, Acer3 null mice on a Sv129:C57BL/6 mixed genetic background were backcrossed to the C57BL/6J genetic background for 16 generations. Animal studies were approved by the Institutional Animal Care and Use Committee at Stony Brook University (Stony Brook, NY, USA).

### Isolation and culture of murine PMs, BMCs and CECs

PMs were isolated from 8-week-old mice as described.^54^ BMCs were isolated from mice at the same age using Ficoll-Paque density centrifugation media (GE Healthcare Life Sciences, Pittsburgh, PA, USA) according to the manufacturer's instructions. CECs were isolated from mouse colons as described.^55^ Briefly, mouse colons were sterilized for 15 min with 0.04% sodium hypochlorite in PBS at room temperature. After being washed with PBS, the colons were incubated for 90 min in PBS containing 3 mM EDTA and 0.05 mM dithiothreitol DTT) at room temperature. The colons were shaken vigorously in PBS to liberate crypts from the submucosa. The liberated crypts were harvested by centrifugation at 400 rpm for 5 min and were digested for 90 min with 0.25% pancreatin (Sigma-Aldrich, St. Louis, MO, USA) at room temperature. CECs released from crypts were collected by centrifugation at 1000  r.p.m. for 5 min, and resuspended in the EDTA/DTT solution. PMs and BMCs were cultured in RPMI 1640 medium containing penicillin, streptomycin and 10% fetal bovine serum in regular tissue culture plates, and CECs were cultured in the same medium in tissue culture plates coated with Matrigel (BD Biosciences, Franklin Lakes, NJ, USA). For LPS stimulation, BMCs were plated at a density of 10^6^ cells per well in six-well culture plates and were immediately treated with 50 ng/ml LPS from *γ*-irradiated *Escherichia coli* 0111:B4 (Sigma-Aldrich) or the Veh (PBS). PMs and CECs were plated at the same cell density in regular and Matrigel-coated six-well plates, respectively, for 60 min before being treated with LPS or PBS.

### Murine model of colitis

To induce colitis, male and female Acer3^+/+^ and Acer3^−/−^ mice (approximately 1 : 1 ratio) at 6 weeks of age were administered on drinking water containing 2.5% w/v DSS (molecular weight, 35 000–50 000; MP Biomedicals, Solon, OH, USA) for up to 7 days. Acer3^+/+^ or Acer3^−/−^ mice of the same sex were randomly placed in different cages with a maximum of four per cage and were housed in the same animal facility room. Mice in control (CTR) group were given regular water during the same period. Mice were examined daily during or after DSS treatment for their weight loss, diarrhea or rectal bleeding. A colitis activity index was expressed as the sum of the scores of stool consistency (0, normal beaded stool; 1, soft stool; 2, diarrhea) and rectal bleeding (0, no bleeding; 1, blood present in stool or anal area), thus, the minimal and maximal index are 0 and 3, respectively. For necropsy, mice were killed after being treated with DSS for 3, 5 or 7 days or 3 days post the 7-day DSS treatment, and colon length and spleen weight were recorded.

### Murine model of CAC

Male and female Acer3^+/+^ and Acer3^−/−^ mice (approximately 1 : 1 ratio) at 6 weeks of age were were injected intraperitoneally with one dose of AOM (10 mg/kg; Sigma-Aldrich). Seven days later, the mice were given DSS (2.5%) in the drinking water for 7 days. Following the DSS treatment, mice were switched to regular water and killed 9 weeks later to evaluate colon length, inflammation and tumorigenesis. Colons were opened to determine tumor numbers and size. Tumor volumes were measured using a caliper and were calculated using the following formula: width^2^ × length.

### Vascular permeability assays

Vascular permeability was examined by EBD extravasation as described^56, 57^ with slight modifications. Briefly, after 5-day acute colitis induction, DSS-treated mice or mice on regular water were injected with 150 *μ*l of 0.5% EBD (Sigma-Aldrich) in sterile saline through the lateral tail vein. At 1 h post EBD injection, mice were killed and colons were collected and flushed with PBS. After drying overnight at 56 °C, colons were weighted and the extravasation of EBD was quantified by spectrophotometric absorbance at 610 nm. EBD concentrations were calculated according to a standard curve of known concentrations of EBD and normalized to tissue weights.

### Intestinal epithelial permeability assays

Intestinal epithelial permeability was determined by FITC-dextran assays as described^23^ with slight modifications. Mice were administered orally with 200 *μ*l of 2 mg/ml FITC-dextran (average molecular weight, 4400; Sigma-Aldrich). Whole blood was collected by cardiac puncture at 6 h after FITC-dextran administration, and serum fluorescent density was determined by fluorometry. FITC-dextran concentrations were calculated according to a standard curve of known concentrations of FITC-dextran.

### Complete blood cell count

After killing, whole blood was collected in EDTA-coated tubes from mice by cardiac puncture, and 20 *μ*l of whole blood were used for CBC in Hemavet Hematology Analyzer (Drew Scientific, Waterbury, CT, USA).

### Histologic analyses

Mouse colons were transected longitudinally. One half of each colon was rolled onto itself as a ‘Swiss roll' and fixed in 4% PFA, and another half was snap frozen in liquid nitrogen and stored at −80 ^o^C. Fixed tissues were embedded in paraffin blocks and sectioned. The tissue sections were stained with hematoxylin and eosin (H&E) or periodic acid–Schiff and Alcian blue (PAS/AB). H&E-stained sections were imaged under an Imager M2 microscope (Zeiss, Thornwood, NY, USA), and crypt damage and inflammation were scored as described by Lynn *et al.*^58^ using a scoring scheme as presented in Supplementary Table S1. The total histology score is the sum of the subscores of epithelial damage and inflammatory infiltration, thus, the minimal score is 0 and the maximal score is 40. In the CAC model, colon tumor grading was performed by a pathologist on H&E-stained sections in a blinded manner.

### Immunohistochemistry

Ki-67 immunostaining was performed as described.^10^ Ki67-positive cells were scored on 20 × objective field of view under an Imager M2 microscope (Zeiss) in a blinded manner. In the DSS-colitis model, Ki67-positive cells per crypt were calculated; and in the AOM/DSS CAC model, percentage of positive cells in tumors were enumerated.

### Terminal deoxynucleotidyl transferase dUTP nick end labeling (TUNEL) assays

Colon sections were prepared as described above and subjected to TUNEL assays as described in our previous studies.^10^ TUNEL-positive cells were enumerated from four random 10 × fields of view in a blinded manner, and positive cells per 10 × field were calculated.

### MPO activity assays

MPO activity in mouse colon tissues was determined using MPO Colorimetric Activity Assay Kits (Sigma-Aldrich) according to the manufacturer's instructions.

### Protein concentration determination

Protein concentrations were determined with bovine serum albumin as a standard using a bicinchoninic acid protein determination kit (Thermo Scientific, Waltham, MA, USA) according to the manufacturer's instructions.

### Western blot analyses

Colon tissues were homogenized, and protein extracts were subjected to western blot analyses as described.^10^ Antibodies used in this study are: proliferating cell nuclear antigen (PCNA) antibody, cleaved caspase-3 antibody, from Cell Signaling, Beverly, MA, USA, and *β*-actin antibody from Santa Cruz, Dallas, TX, USA. Protein band density was measured by densitometry and analyzed using the NCBI software Image J (Bethesda, MD, USA).

### Alkaline ceramidase activity assays

Colon tissues were homogenized on ice with an electric tissue tearor (Biospec Products, Bartlesville, OK, USA) in buffer A (25 mM Tris-HCl, pH 7.4, 150 mM NaCl and 0.25 M sucrose) supplemented with a protease inhibitor mixture (Roche, Indianapolis, IN, USA). After brief sonication, tissue homogenates were centrifuged at 1000 *g* at 4 ^o^C for 5 min, and the resulting supernatants were centrifuged at 100 000 *g* at 4 ^o^C for 45 min to sediment all cell membranes, which were resuspended in buffer B (25 mM Tris, pH 7.4, 5 mM CaCl_2_ and 150 mM NaCl) and homogenized by sonication. Membrane homogenates (20 *μ*g protein per tissue) were measured for alkaline ceramidase activity using NBD-C_12_-PHC as a substrate as described.^10^

### Liquid chromatography tandem mass spectrometry

Colon tissues were collected and washed with buffer C (25 mM Tris-HCl buffer, pH 7.4, 150 mM NaCl). The colon tissues were homogenized on ice as described earlier in buffer D (25 mM Tris-HCl, pH 7.4, 150 mM NaCl, 1 mM EDTA and 1 mM EGTA). Tissue homogenates (2 mg protein per sample) or intact cells were subjected to lipid extraction and LC-MS/MS analyses for sphingolipids as described.^10^ Amounts of sphingolipids were normalized to protein concentration.

### RNA extraction and quantitative PCR (qPCR)

Total RNAs were isolated from fresh tissues or cells using a RNeasy mini kit (QIAGEN, Valencia, CA, USA). RNAs were reversely transcribed into cDNAs and mRNA levels of *Acer3*, *Acer2*, *Tnf-α*, *Il-6*, *Il-23a*, *Il-1β* *or β-*actin (as reference gene) were determined by qPCR as described.^10^ Primers were listed in Supplementary Table S2.

### Online data mining

Graphpad Prism 5.0 (La Jolla, CA, USA) was used to analyze ACER3 expression in the NCBI GEO data sets of inflammation-related studies.

### Data analysis

Statistical analyses were performed using the Student's *t*-test or two-way AVONA using Graphpad Prism 5.0. *P*-value <0.05 were considered significant.

## Abbreviations

Acer3: alkaline ceramidase 3
CECs: colonic epithelial cells
GEO: NCBI Gene Expression Omnibus
LPS: lipopolysaccharides
SPT: palmitoyltransferase
CerSs: (dihydro)ceramide synthases
SMases: sphingimyelinases
SPH: sphingosine
PMs: peritoneal macrophages
UC: ulcerative colitis
IBD: inflammatory bowel disease
DSS: dextran sulfate sodium
CAC: colitis-associated colon cancer
ULCCs: unsaturated-long-chain ceramides
BMCs: blood mononuclear cells
S1P: sphingosine-1-phosphate
