# Supplementary figures and images for: Alkaline ceramidase 3 deficiency aggravates colitis and colitis-associated tumorigenesis in mice by hyperactivating the innate immune system
Source: Cell Death Dis. 2016 Mar 3;7(3):e2124–. doi: 10.1038/cddis.2016.36 (PMC4823937; doi:10.1038/cddis.2016.36)

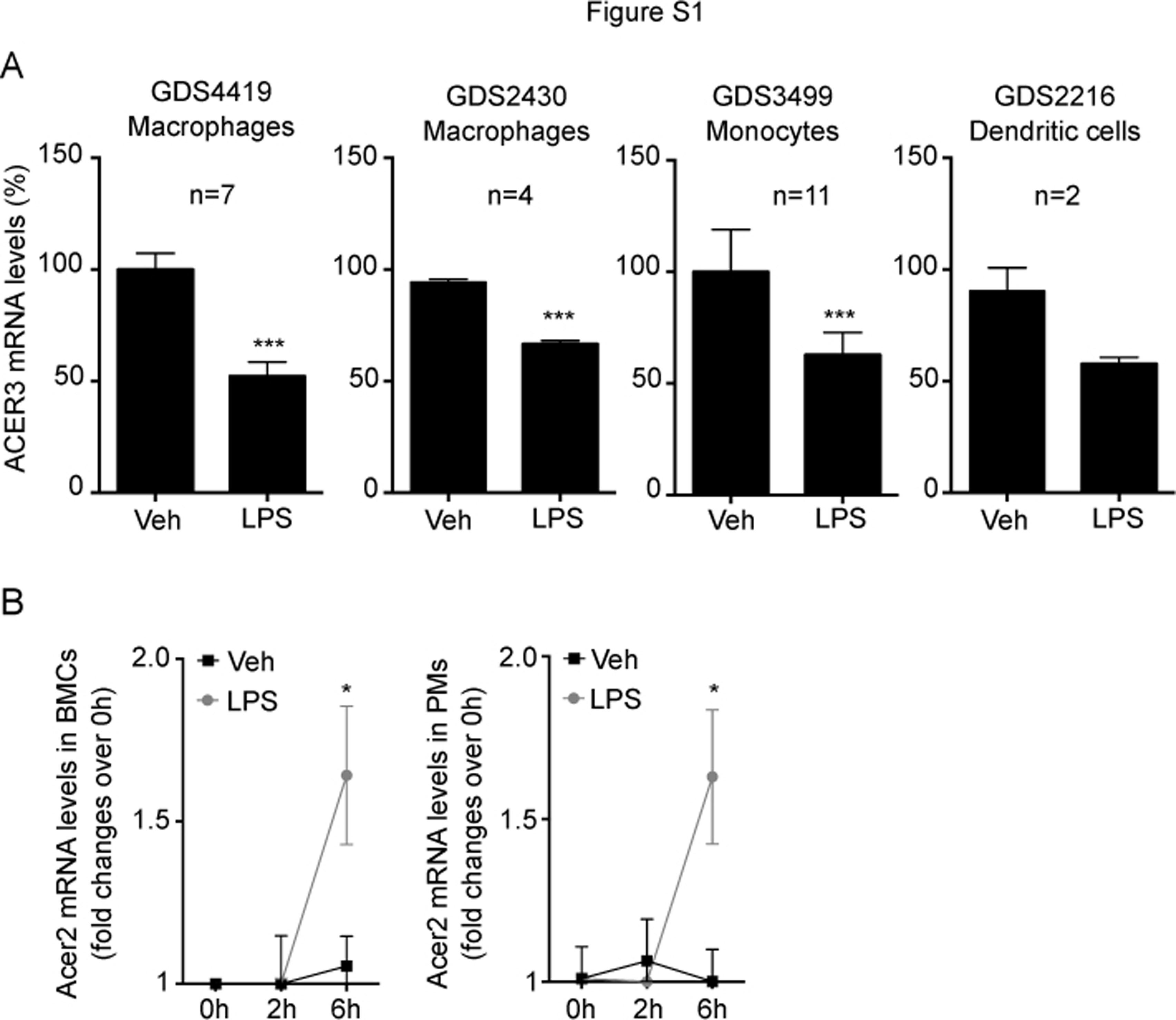

Supplement: Supplementary Figure 1 [file cddis201636x1.tif]

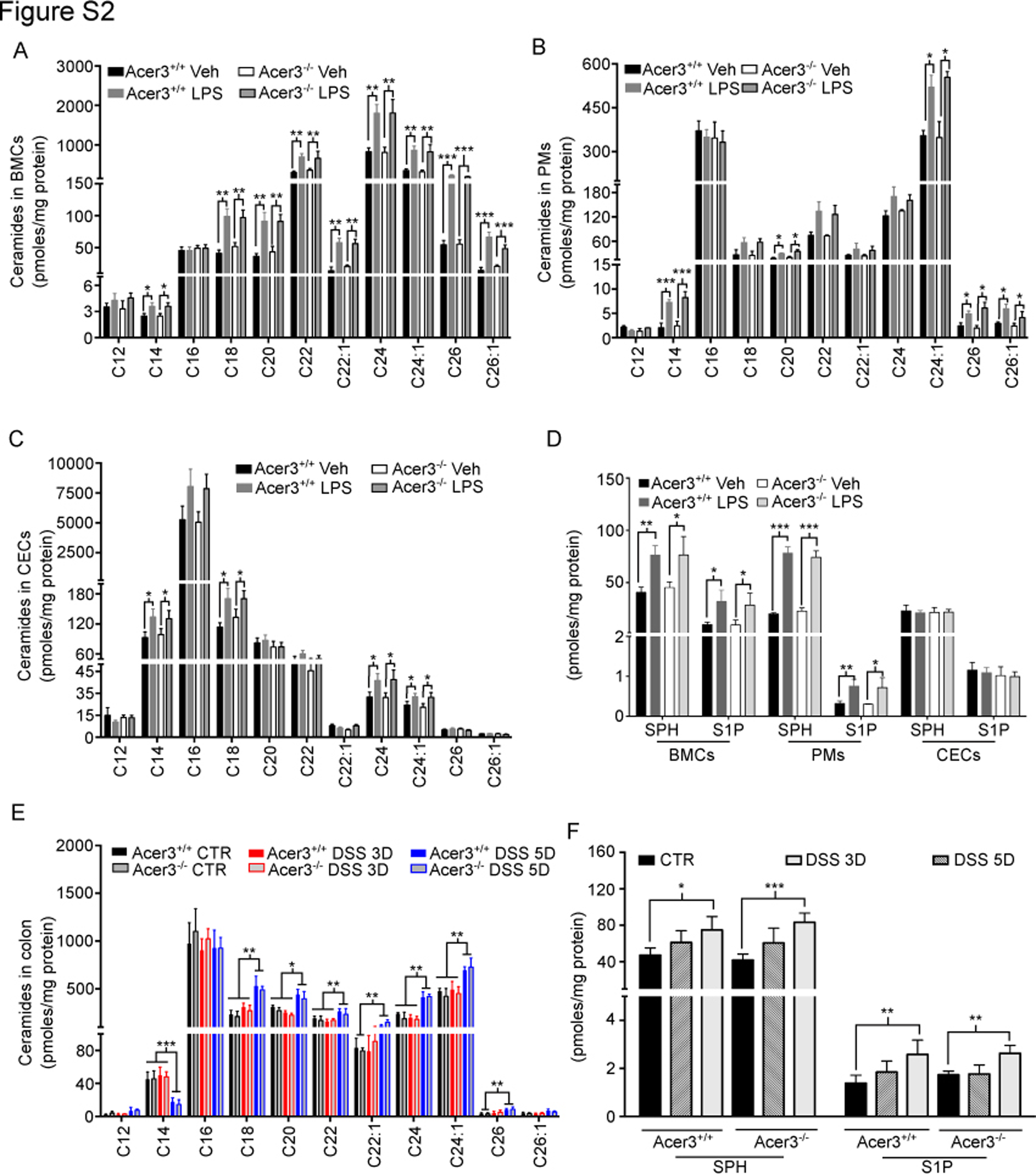

Supplement: Supplementary Figure 2 [file cddis201636x2.tif]

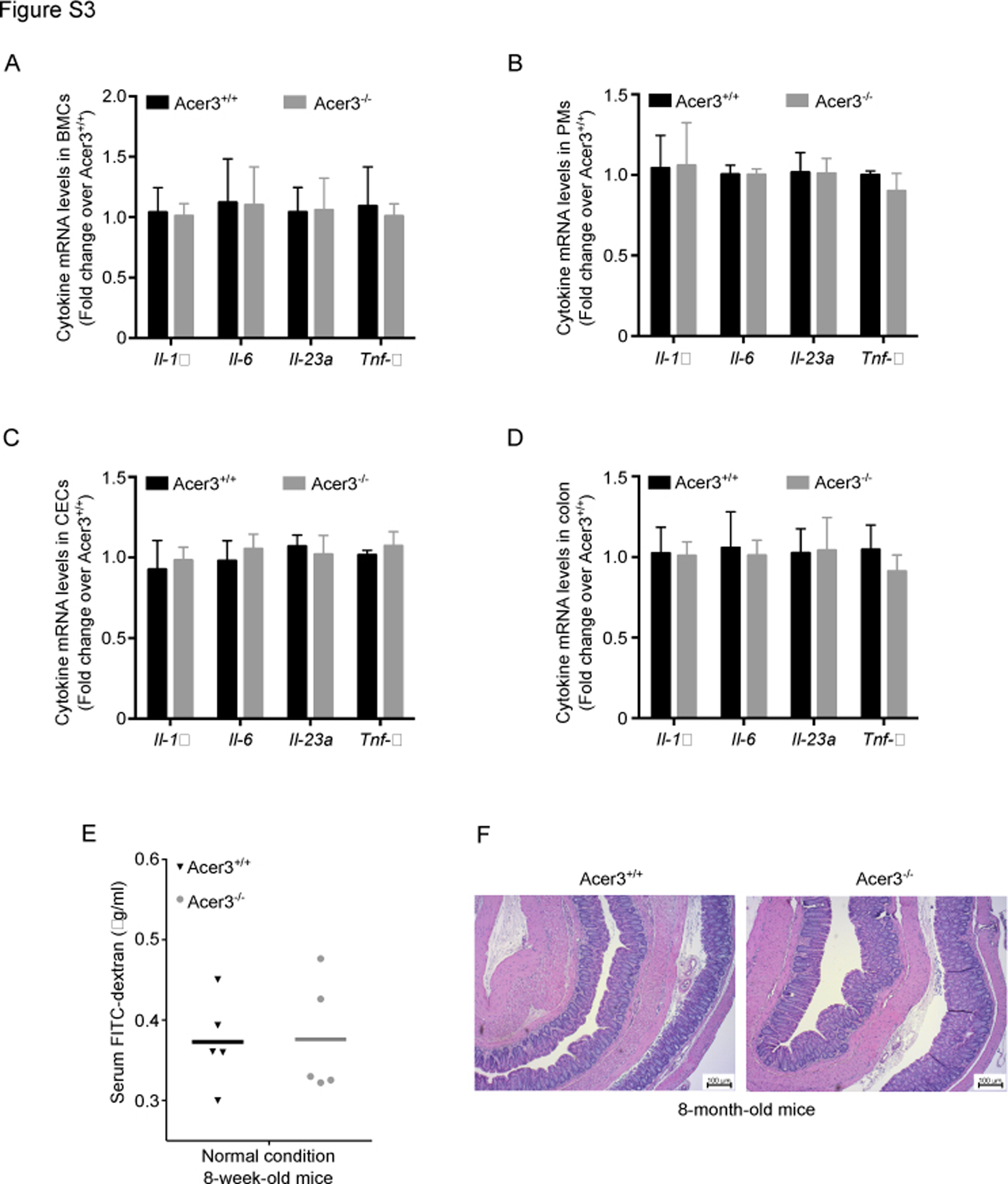

Supplement: Supplementary Figure 3 [file cddis201636x3.tif]

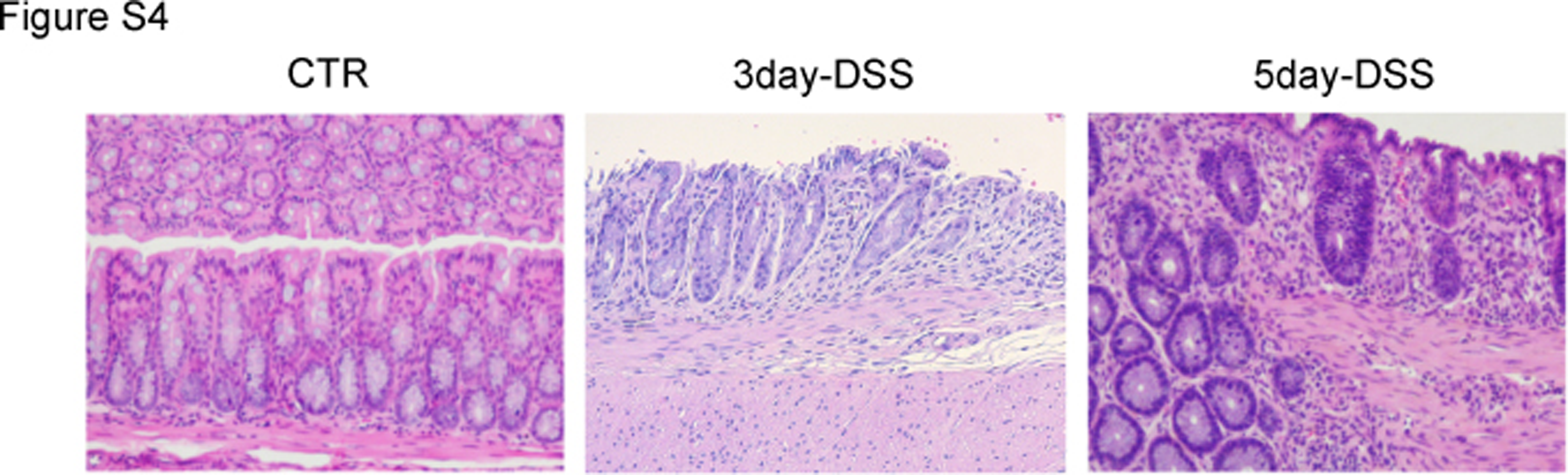

Supplement: Supplementary Figure 4 [file cddis201636x4.tif]

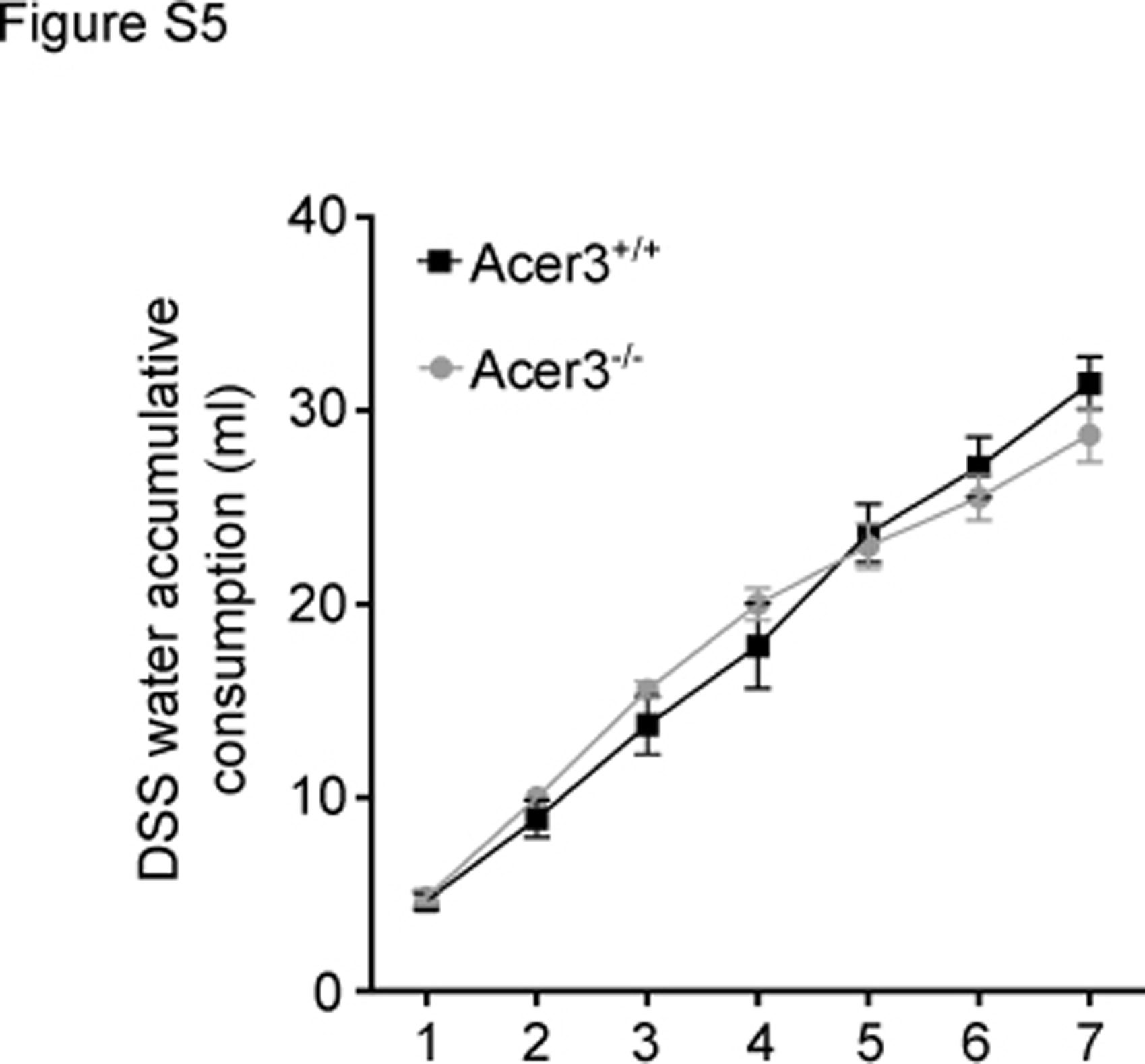

Supplement: Supplementary Figure 5 [file cddis201636x5.tif]

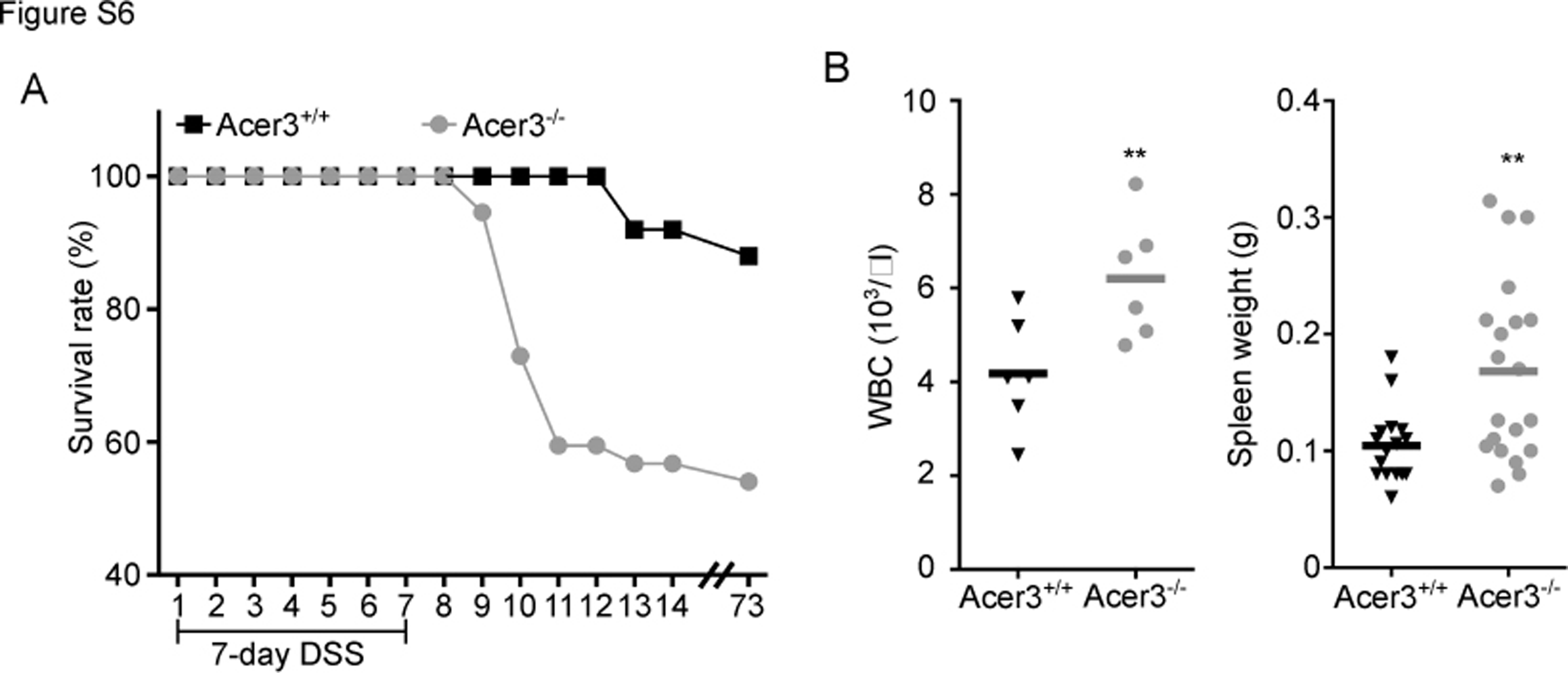

Supplement: Supplementary Figure 6 [file cddis201636x6.tif]

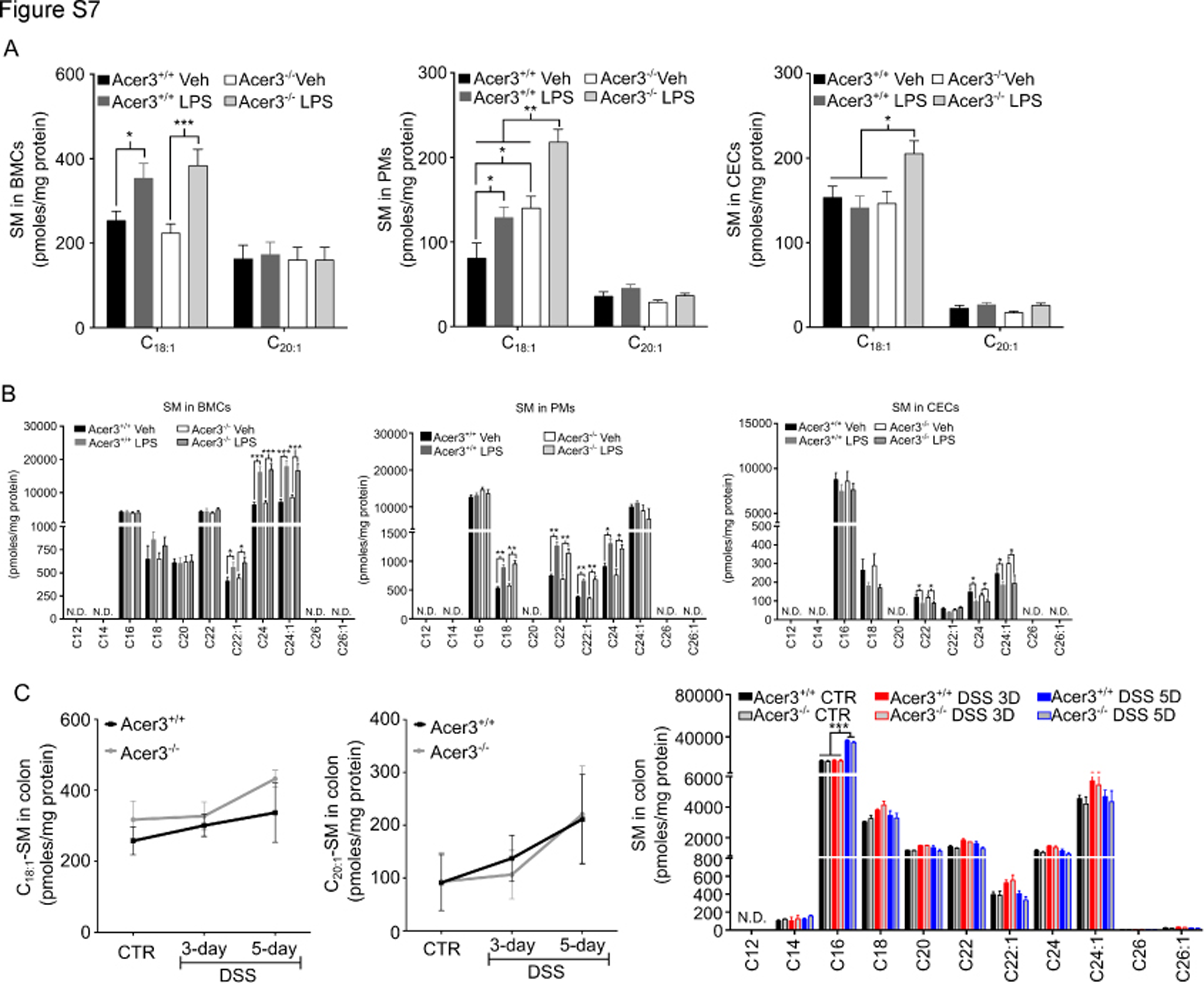

Supplement: Supplementary Figure 7 [file cddis201636x7.tif]
