# Supplementary material for: Alkaline ceramidase 3 deficiency aggravates colitis and colitis-associated tumorigenesis in mice by hyperactivating the innate immune system
Source: Cell Death Dis. 2016 Mar 3;7(3):e2124–. doi: 10.1038/cddis.2016.36 (PMC4823937; doi:10.1038/cddis.2016.36)
Supplement: Supplementary Information [file cddis201636x8.doc]

**SUPPLEMENTARY INFORMATION**

**Figure S1. LPS decreases Acer3 mRNA levels in human immune cells and increases Acer2 mRNA levels in mouse immune cells.**

**A**, the datasets GDS4419 ^11^, GDS2430 ^12^, GDS3499^13^, and GDS2216 ^14^ in the NCBI GEO database were analyzed using Graphpad Prism 5 for LPS-induced percent changes in the *Acer3* mRNA levels in human macrophages, monocytes, and dendritic cells, respectively, in comparison to Veh.

**B**, BMCs and PMs were isolated from C57BL/6 mice as described in Materials and Methods and stimulated with LPS as in Figure 1. Total RNAs were isolated from the above treated cells and Acer3 mRNA levels were measured by quantitative PCR (qPCR) analyses. Data are expressed as percent changes of *Acer2* mRNA levels in LPS-treated cells over those in Veh-treated cells.

Data in A represent mean values ± SEM. Data in B represent mean ± SD from n=3. **p*<0.05*.*

**Figure S2. LPS and DSS alter ceramides in cells and colon tissues, respectively.**

**A**-**D,** BMCs (A), PMs (B), and CECs (C) from Acer3^+/+^ and Acer3^-/-^ mice were treated with 50 ng LPS or PBS for 6 h before they were subjected to LC-MS/MS for the levels of ceramides. In the above cells, the levels of SPH and S1P were also determined by LC-MS/MS (D).

**E** and **F**, colon tissues were collected from Acer3^+/+^ and Acer3^-/-^ mice on DSS or regular water for 5 days and the levels of ceramides (E), SPH and S1P (F) were determined by LC-MS/MS.

Data in A, B, C and D represent mean values ± SD from 3 independent experiments, error bar indicates SD. Data in E and F represent mean values ± SD, error bar indicates SD, n=4-6, **p*<0.05*, **p*<0.01, ****p*<0.001.

**Figure S3. Acer3 deficiency does not cause inflammation or affect intestinal epithelial permeability and integrity in mice at baseline.**

**A-D,** inflammatory cytokine levels were determined by qPCR in mouse BMCs (A), PMs (B), CECs (C), and colon tissues (D) from 6-week-old Acer3^+/+^ or Acer3^-/-^ mice.

**E**, intestinal epithelial permeability in Acer3^+/+^ and Acer3^-/-^ mice at 8 weeks of age was determined using FITC-dextran.

**F**, colons from 8-month-old Acer3^-/-^ mice were histologically examined for the structural integrity of colons.

Data in A, B, C, and D represent mean ± SD, n=5. The images in F are representative results of five pairs of mice.

**Figure S4. Colitis induction in WT mice.**

WT mice were on regular drinking water (CTR group) or water with 2.5% DSS for 3 or 5 days. Colon tissue were processed and stained by H&E staining, images of distal colon were taken. Pathologic manifestations, including inflammatory cell infiltration and epithelial damage, were histologically monitored. Histological analyses showed that inflammation occurred in part of the distal colon after 3-day DSS treatment, and then extended to the whole distal colon and part of the middle colon after 5-day DSS treatment, whereas CTR mice fed with regular water had normal intestinal epithelia,

Images represent results from one of four mice.

**Figure S5. Acer3 deficiency does not affect DSS water intake.**

Acer3^+/+^ and Acer3^-/-^ mice were on DSS-containing or regular water, and water intake were monitored. Data represent mean ± SD, n =10.

**Figure S6. Acer3 deficiency increases mortality and systemic inflammation in a murine model of CAC.**

**A**, CAC was induced in Acer3^+/+^ and Acer3^-/-^ mice. Mouse survival rate was monitored until 80 days after AOM injection (73 days after 7-day DSS treatment), n=20-35.

**B**, the above mice were euthanized at 80 days after AOM injection, and blood was collected for CBC and mouse spleens were weighed. ***p*<0.01.

**Figure S7. Acer3 deficiency enhances the elevation of C_18:1_-SM in LPS-stimulated mouse PMs but not in DSS-treated colon tissues.**

**A** and **B**, BMCs, PMs, and CECs of Acer3^+/+^ and Acer3^-/-^ mice were plated as described in Materials and Methods and treated with 50 ng LPS or PBS for 6 h before they were subjected to LC-MS/MS for the levels of unsaturated-long-chain SM (A) and other SM species (B).

**C**, colon tissues were collected from Acer3^+/+^ and Acer3^-/-^ mice on DSS or regular water for 3 or 5 days and the levels of SM were determined by LC-MS/MS.

Data in A and B represent mean ± SD, n=3. Data in C represent mean ± SD, n=4-6, *p<0.05, **p<0.01, ***p<0.001. N.D. Not detected.

**Table S1 Histological scoring system for DSS-induced colitis.**

| Manifestation Scored | Score | Description |
| --- | --- | --- |
| Inflammation Severity | 0 | None |
|  | 1 | Mild |
|  | 2 | Moderate |
|  | 3 | Severe |
| Inflammation Extent | 0 | None |
|  | 1 | Mucosa |
|  | 2 | Mucosa and Submucosa |
|  | 3 | Transmural |
| Crypt Damage | 0 | None |
|  | 1 | 1/3 of Crypt Damaged |
|  | 2 | 2/3 of Crypt Damaged |
|  | 3 | Crypt lost, surface epithelium intact |
|  | 4 | Crypt lost, surface epithelium lost |
| Percent of Involved Area | 0 | 0% |
|  | 1 | 1-25% |
|  | 2 | 26-50% |
|  | 3 | 51-75% |
|  | 4 | 75-100% |

**Table S2. Primers used in real-time PCR analysis.**

| Gene | Primer sequence |
| --- | --- |
| *β-atcb* | 5’-GATGTATGAAGGCTTTGGTC-3’ and 5’-TGTGCACTTTTATTGGTCTC-3’ |
| ** | 5’-GTGTGGCATATTCTCATCTG-3’ and 5’-TAAGGGACACCAATAAAAGC-3’ |
| *Acer3* | 5’-ATGCTCATAGGTCTGTTCTC-3’ and 5'-AGTGGTTATAGTTACCAGGC-3’ |
| *Il-1β* | 5’-GGATGATGATGATAACCTGC-3’ and 5’-CATGGAGAATATCACTTGTTGG 3’ |
| *Il-6* | 5’- AAGAAATGATGGATGC TACC-3’and 5’-GAGTTTCTGTATCTCTCTGAAG-3’ |
| *Il-23a* | 5’-AATAA TGCTATGGCTGTTGC-3’ and 5’-CTTAGT AGATTCATATGTCCCG-3’ |
| *Tnf-α* | 5’-ctatgttctcagcctcttctc-3’ and 5’-CATTTGGGAACTTCTCATCC-3’ |
